# Hours based scheduling in neonatology: a practical approach

**DOI:** 10.1038/s41372-025-02332-y

**Published:** 2025-06-19

**Authors:** Annemarie Stroustrup, Patrick J. McNamara, Trent E. Tipple, Satyan Lakshminrusimha

**Affiliations:** 1 Northwell Health; Division of Neonatology, Cohen Children’s Medical Center, Department of Pediatrics, Zucker School of Medicine at Hofstra/Northwell, New Hyde Park, NY USA; 2 Division of Neonatology, University of Iowa Children’s Hospital, University of Iowa, Iowa City, IA USA; 3 Section of Neonatal-Perinatal Medicine, Department of Pediatrics, University of Oklahoma College of Medicine, Oklahoma City, OK USA; 4 Department of Pediatrics, University of California at Davis, Sacramento, CA USA

**Keywords:** Health occupations, Health services

## Abstract

No standard work assignment exists for practicing neonatologists in the United States. Unlike other types of care providers in the neonatal intensive care unit who work under standard shift or work hour expectations, attending neonatologist staffing models vary significantly by job task, shift length, in-person or remote coverage responsibility, ambulatory or inpatient practice, and total clinical versus non-clinical commitments among other variables. Due to the diversity of clinical and non-clinical responsibilities of practicing neonatologists, transparent and equitable staffing models can be challenging to design and execute. We present a flexible approach to neonatologist scheduling that has been implemented effectively at two academic medical centers with multiple sites and types of neonatology clinical practice. This model allows for clear delineation of time dedicated to a variety of clinical and non-clinical activities to allow both clinical and administrative leadership clarity on the full range of professional responsibilities of a practicing neonatologist.

## Introduction

The pediatric subspecialty of neonatology was born with the death of the sitting president John Fitzgerald Kennedy’s premature infant son on August 9, 1963. Patrick Bouvier Kennedy was delivered at 34 weeks’ gestation and lived only two days before succumbing to respiratory distress syndrome. At that time, approximately 18/1000 newborns in the United States died during the first postnatal month after birth. Although attempts to develop care practices specific for ill neonates had been made earlier in the century, federal funding for and national interest in the care and diseases of newborns increased dramatically with this very public death due to prematurity. Neonatal care has improved dramatically through subsequent decades, due to enhanced staffing models, improved understanding of perinatal physiology, and evidence-based practice. Radical improvements in the care of prematurity, including necessary miniaturization of diagnostic and therapeutic equipment, the discovery of surfactant, development of intravenous nutrition and specialty enteral formulas, and collaborative improvements in maternal care, such as the introduction of antenatal steroids and magnesium administration specifically to improve child outcomes. Today, the neonatal death rate is <6/1000 births from all causes [1], and prematurity as early as 22 weeks’ gestation is survivable [2]. A 1-kg infant who was born in 1960 had a mortality risk of 95%, but by 2000 had a 95% probability of survival [3].

Advancements in neonatal care paralleled the growth in the number of dedicated neonatology subspecialists. Fellowship training programs in Pediatric Cardiology, developed in the late 1950s, had demonstrated the benefit of additional training beyond a General Pediatric Residency for those interested in specific areas of pediatric care. By the early 1970s, it was clear that subspecialty training in neonatology, or neonatal-perinatal medicine, was also warranted. In 1975, the American Board of Pediatrics offered the first certification examination in Neonatal-Perinatal Medicine, certifying 357 neonatologists. Fellowship training programs multiplied over time, allowing for an increasing number and availability of board-certified neonatologists at any given center. In recent bi-annual testing cycles, 400–600 neonatologists passed their initial board certification (586 in 2022) [4]. To date, 7871 physicians have been board-certified in Neonatal-Perinatal Medicine [5], with most remaining in practice. By 2023, there were 5319 neonatologists maintaining certification in the United States [6].

The neonatologist workforce in the United States is unique compared to other pediatric subspecialties in several ways. *First*, neonatologists are less likely to hold full-time academic appointments or have dedicated research time. *Second*, they are more likely to be female (77% versus 70% for other pediatric specialties), international medical graduates (37% versus 26% for other pediatric specialties), and work >60 h per week (28% versus 23% for other pediatric specialties) [6].

As the practice of neonatology and the neonatologist workforce have evolved, staffing models and expectations have changed dramatically. Driven by scarcity, neonatology began with in-person neonatologist staffing primarily on weekdays only. At most institutions, a limited number of neonatologists would typically share daytime coverage as “weeks of service” and cover nights and most weekend time remotely as “home call” with trainees, nursing, and/or advanced practice staff executing care plans conveyed over the phone. Schedules were created by dividing annual coverage needs as weeks of service amongst an institution’s available neonatologists, without consideration of the time or effort required. This was possible, in part, due to significant clinical independence given to early-stage trainees and/or advanced practice staff, far beyond what is considered acceptable today.

Over time, as patient volume and acuity grew, clinical quality and supervision expectations rose. As the pool of trained neonatologists increased, high-intensity, often in-person neonatologist presence has increasingly become the norm. In parallel, clinical outcomes for ill neonates have improved dramatically [7, 8]. Currently, there remains significant variability in the determination of neonatologists clinical schedules, including what clinical work is included in “scheduled” time [9, 10]. In contrast with some other pediatric subspecialties, there is no agreed-upon standard clinical full-time equivalent (cFTE) for attending neonatologists. This results in high variability in the interpretation of the definition of cFTE. Instead, cFTE is often driven by departmental needs, institutional mandates, and leadership priorities, often to the detriment of provider well-being.

In this report, we share one approach to scheduling neonatologist professional time that is easily adaptable to a variety of institutional requirements and practice settings, with intentionally created flexibility to include those with exclusively clinical responsibilities and those with additional academic, administrative, operational, or educational protected time. This approach is readily scalable for small or large groups, covering any number of clinical roles or practice sites. Most importantly, the presented scheduling model provides the opportunity for transparency and equity in neonatologist staffing and can serve as a template for neonatology leaders who desire to develop national standard practice models.

## Methods

Neonatologists are responsible, whether physically at the bedside or as a remote supervisor, for a variety of care types in a variety of locations. In addition to management of the intensive care unit neonatologists provide ambulatory care for prenatal consults and in follow-up programs; telehealth and transport services; neonatal resuscitation on the labor floor; and specialized services including neonatal cardiac intensive care, neuro intensive care, hemodynamic services, point of care ultrasound within and outside of the neonatal intensive care unit (NICU; Supplemental Table 1). Modern NICU coverage models should include all clinical care, whether entirely in the hospital or a mix of in-person and remote coverage from home. The following steps may be utilized by neonatology leadership to develop an operational framework for clinical work adjudication

### Step 1: Determine the specific roles covered by the neonatologist group and assign a time-based value for each type of coverage

We and others [9] favor a model where all hours worked are the unit of measure for scheduling. Weekday rounding on a high-acuity team may require 50–60 h in a week to account for time in direct patient care, supervision of a multidisciplinary team, participation in clinical/operational conferences, documentation, and billing. Shifts in a lower acuity unit may be valued at the exact shift length, as documentation can be completed within the shift, and time for handoff is minimal. Clinic coverage may require a standard 4-to-5-h ambulatory session. Overnight hours may be valued as the exact shift length, assigned a premium for undesirability (shift differential or hazard allowance, e.g., 1 h of coverage is assigned 1.1 or 1.5 h of credit), or assigned a fraction of an hour if coverage is provided remotely (e.g., 1 h of home call is 0.5 or 0.3 h of credit). Non-clinical responsibilities can also be valued as expected hours needed for role or task completion.

### Step 2: Determine the professional availability of each neonatologist

It is important to refer to institutional benefits packages to ensure compliance with contractual paid time off (PTO), guaranteed holiday time, continuing medical education (CME), family medical leave (FMLA), and any other protected time. At some institutions, these benefits are universally applied while at others they may vary by job class, full- or part-time status, or years of service to the organization.

### Step 3: Define the number of expected working hours in a week

In some cases, this is standardized at the institutional or departmental level; of note, 40–50 working hours in a week is a common standard for physicians in the United States. A neonatologist’s professional availability can then be calculated as:

$$({{\rm{Total}}}\; {{\rm{working}}}\; {{\rm{time}}}\; {{\rm{in}}}\; {{\rm{a}}}\; {{\rm{year}}}){{{-}}}({{\rm{guaranteed}}}\; {{\rm{holiday}}}\; {{\rm{time}}}){{{-}}}({{\rm{PTO}}}){{{-}}}({{\rm{CME}}})$$

**Fig. 1:**
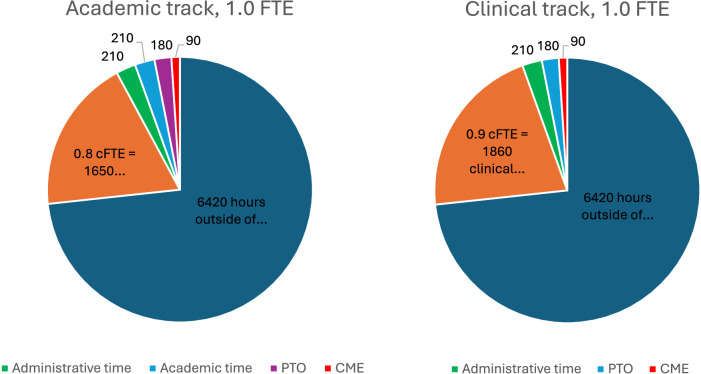
Hours-based scheduling model. Example time allotments demonstrating all hours in a year. Blue hours are non-working hours. Working hours are divided between paid time off (PTO), continuing medical education (CME), clinical, and academic missions.

In practice, it is often easiest to complete the above calculation in weeks (as human resource policies often specify weeks of PTO or CME) and then convert the time availability to hours based on the specified number of expected working hours in a week. Annual working hours can then be further divided into a clinical time allotment (cFTE), excluding time protected for administrative tasks, research or other scholarship, teaching and mentorship, clinical operations or quality, or any other responsibilities outside of clinical care. A standard schedule for a full-time clinician would typically allow at minimum 10% non-clinical “protected” time for required administrative tasks, including annual trainings, participation in clinically oriented meetings such as morbidity and mortality conferences, clinical quality and/or performance improvement meetings, faculty meetings, and the like. A standard schedule for a full-time academic neonatologist would include the necessary administrative time and, at minimum, an additional 10% non-clinical protected time for scholarship and mentorship. Protected time for additional roles (medical director, division director, fellowship program director) or outside funding (research grants, teaching dollars) should also be included in the model. Based on this modeling, most institutions will define a working year as 2000–2100 h, such that a full-time clinician (0.9 cFTE) will work approximately 1800–1900 clinical hours and an academic neonatologist (0.8 cFTE) will work approximately 1600–1700 clinical hours. This method is commonly referred to as the CARTS (clinical, administrative, research, teaching and service) model [11, 12] or “one-minus” methodology, where 1.0 full-time equivalent (FTE) is defined, and all other non-clinical activities and responsibilities are subtracted to derive the clinical cFTE (Fig. 1). Although commonly used for ambulatory specialties, application to hospital-based specialties with night and weekend commitments may also be appropriate in many situations and provides the possibility to standardize across varied roles and institutions. Physicians working part-time have proportional decreases in both clinical and non-clinical FTE allotments. Of note, all work hours are equally weighted irrespective of whether they are relative value unit (RVU) generating. This is particularly important in a 24/7 specialty such as neonatology, where RVUs are largely generated during daytime hours while the most burdensome times for the physician team to cover are nighttime hours with relatively little opportunity for RVU generation.

## Results

Table 1 is a spreadsheet that was developed over time by neonatology leadership at two institutions to schedule clinical and non-clinical time for a neonatology group of varying size and scope. Development occurred at two academic health systems, each with multiple NICUs of varying levels of care (II, III, and IV), with average unit daily census varying from 2 to 54. Units included inborn and outborn patients. Faculty size varied from 12 to 42 neonatologists across the period of spreadsheet development. This model accommodates various “tracks” (academic, non-academic, nocturnist, etc.), coverage models (in-house, ambulatory, “home call”), part-time providers, and group sizes. In addition, the underlying table structure is easily scalable.

**Table 1:** Template for hours-based scheduling [20].

Faculty member	Track (1 = Academic; 2 = clinical; 3 = nocturnist)	PTO (wks)	FTE	Level III/IV service weeks	Level IIB service weeks	Level IIA 24-h shifts	Level IIA day shifts	Level IIA night shifts	v	v	v	v	v	v	v	v	v	Follow- up clinic days	Ambulatory prenatal consult hours	Clinical hours	Total # nights	Total # weekend shifts	cFTE (assuming 1.0 FTE)	Non-clinical FTE (assuming 1.0 FTE)	Reason for non- clinical FTE	Goal cFTE hours by track and effort	Total goal work hours for 1.0 FTE
									l I I	l I	l I	l I	l I I	l I I	l I I	l I I	l I I										
				50	45	25.2	12	13.2										8	0								
Senior academic faculty member	1	4	1	10	0	9	0	0	0	4	2	4	3	0	0	0	4	6	0	1373	36	13	0.84	0.16	Medical Director	1627	2034
Junior academic faculty member	1	4	1	8	8	0	0	0	#	0	0	0	0	0	0	#	0	0	0	1640	40	20	1.01	0.01		1627	2034
Primary clinician	2	4	1	8	0	24	0	2	0	0	0	2	0	0	0	#	0	13	60	1811	52	18	0.99	0.01		1831	2034
Primary clinician	2	3.2	0.8	6	0	25	0	0	0	3	0	4	7	0	0	7	0	10	60	1490	39	18	0.8	0.2		1863	2070
Nocturnist	3	3.6	0.6	0	0	0	0	0	0	0	#	0	0	0	0	0	0	0	0	1091	62	13	0.59	0.41		1847	2052
Nocturnist	3	6	1	0	0	0	0	0	0	0	#	0	0	0	0	0	0	0	0	1725	98	20	0.99	0.01		1750	1944

Total scheduled coverage	32	8	58	0	2										29	120	4824	229	5.22	0.80
Annual coverage need	312	52	252	194	194	#	#	#	#	#			#	#	104	200	7757	1204		
Uncovered	280	44	194	194	192										75	80	6117	975		
Moonlighting/per diem			192	2													6549	956		

Table structure:

(1) Full-time effort at our institution is calculated as follows:

a. Weeks of work: (52 weeks in a year) – (1.8 weeks holiday time) – (4 or 6* weeks PTO) – (1 week CME). For most faculty, this is 52-1.8-4-1 = 45.2 weeks of work.

b. Our institution expects 45 h of work each week from physicians. For most faculty, this means 45.2 weeks of work × 45 h/week = 2034 h of work expected each year.

c. These hours of work can be dedicated to clinical care, administration, teaching, research, or scholarship, or some combination of these and/or other institutionally approved activities.

d. We established that FTE allotments are accurate to the tenth decimal place (not necessarily to the hundredth decimal place).

(2) Each faculty member is listed in column A.

(3) The faculty member’s career track is represented in column B.

a. At our institution, neonatologists are on an “Academic”, “Clinical”, or “Nocturnist” track.

i. All neonatologists have 0.1 FTE protected non-clinical effort to complete administrative requirements/duties. These include attendance at required meetings, activities towards maintenance of certification, clinical documentation done outside of clinically scheduled time, etc.

ii. Academic neonatologists have, at minimum, an additional 0.1 FTE protected non-clinical effort for scholarly work and teaching.

iii. Additional leadership and/or administrative responsibilities (i.e., medical director, fellowship program director, quality director, etc.) incur additional protected non-clinical effort to allow for role responsibilities.

(4) The annual allotted paid time off (PTO) populates column C.

a. At our institution, most full-time physicians are awarded 4 weeks PTO.

b. At our institution, many full-time physicians with greater than 20 years of service to the organization are rewarded with 2 weeks additional PTO.

c. PTO is pro-rated for the overall level of effort (i.e., part-time workers receive partial PTO).

(5) The faculty member’s TOTAL FTE populates column D.

a. In the templated example, we have 2 part-time and 4 full-time neonatologists.

(6) Each type of clinical coverage or non-clinical commitment is represented in row 2.

a. The neonatologists in this example table cover one level IV with 4 weekday rounding teams, one level III with 2 weekday rounding teams, one level IIB with a single weekday rounding team, and one level IIA NICU with a single neonatologist providing 24/7 in-house coverage. We also staff an ambulatory follow-up clinic and an ambulatory/telehealth prenatal consult service.

b. Additional roles/responsibilities can be added or removed as needed.

(7) The number of hours credited for each commitment populates row 3.

a. In the templated example, our unit of measure is hours.

b. In our templated example, all overnight or weekend commitments are valued at 10% more than the actual hours assigned to the “shift”. This is our group’s day/night differential (some groups may not have a differential, may provide a higher differential value to night and weekend hours, or may value those undesirable shifts with additional pay rather than time credit).

c. Our template does not include “home call” as our coverage model is fully in-house. Any home call commitment could be assigned an appropriate hour-based value agreed upon by the group (typically something less than 1:1; for example, a home call hour could be valued at 0.3–0.5 h).

(8) Each faculty member’s time commitment is represented on a separate row in the table, with the number of each type of “shift” represented in the appropriate column.

(9) Summary data delineating total clinical hours worked, distribution of shifts, and clinical versus non-clinical effort for each faculty member are presented in columns V through AC.

(10) Summary of service staffing needs and open shifts are presented in rows 11–14, columns B through W.

a. Row 11 tabulates the number of shifts or weeks of service covered by the staff (i.e., the sum of rows 4 through 9).

b. Row 12 tabulates the number of shifts or weeks of service or each type that need to be covered in a year.

i. For example, cell E12 provides the number of weeks of weekday service that need to be staffed in our level III and IV NICUs. As 4 neonatologists round every weekday at our level IV and 2 neonatologists round every weekday at our level 2, this is (4 neonatologists × 52 weeks) + (2 neonatologists × 52 weeks) = 312 weeks of service requiring individual neonatologist coverage.

c. Row 13 tabulates the number of shifts or weeks of service that are currently uncovered (i.e., row 12-row 11). As this example only includes schedules for 6 faculty members for a service that would need approximately 30 faculty to cover, there are a lot of uncovered shifts.

d. Row 14 provides the number of shifts to be covered by moonlighting or per diem work, i.e., shifts to be covered beyond the clinical time commitment of employed neonatologists. At our institution, we cover level II 24-h shifts and night and weekend shifts at the level III and level IV NICUs as moonlighting, paid at an hourly rate above regular guaranteed compensation. This row is very helpful for anticipatory budgeting.

* See point #4 for further clarification on PTO allotment.

The staffing model was developed initially in 2019, with implementation in 2020–2021. Major revisions to the model were not required. The flexibility of the modeling approach was highlighted by the ease of updating for new clinical service obligations/roles, additional neonatologists joining the team, adjustments in effort for leaves of absence, and the addition of additional weighting of nights and weekends over subsequent years. Faculty appreciated the transparency and predictability of scheduling. Hospital administrators appreciated the objectivity brought to discussions about appropriate staffing levels, reflected both by number and the total effort of neonatologists. Specifically, the answer to requests for additional neonatologist FTE could be easily justified. Neonatology leadership appreciated the ease in predicting moonlighting/per diem budgets and the ease in making adjustments with staffing changes, such as neonatologists transitioning from full to part-time or taking leave of absence.

## Discussion

The template described in this manuscript is one example of a transparent scheduling model in current use in large, multi-hospital neonatology practice. It moves beyond the traditional neonatology scheduling model where the credit for clinical work was solely adjudicated based on “weeks of service”, to recognize night call and other forms of clinical care provision as meaningful clinical effort. Examples of inpatient and ambulatory care, low-acuity and high-acuity care, subspecialty team coverage, varying shift lengths, a variety of professional tracks, and both full- and part-time physicians are included. The model can be adapted to specific institutional benefit requirements, including holiday, PTO, FMLA, and CME requirements that may vary by person, job class, or institution. It can also be adapted to include any type of work for which an hourly allotment can be assigned, including remote supervision, telehealth, and non-clinical professional obligations.

Neonatology practice has evolved dramatically since its origins in the 1950s and 1960s. The chronicity, complexity, and clinical acuity of patients in the NICU have increased over the last two decades. The neonatologist workforce has also grown and evolved, such that staffing models must be developed to meet clinical quality and workforce quality of life needs rather than solely trying to maximize the reach of scarce expertise [13]. Staffing models that do not explicitly credit all clinical and non-clinical neonatologist responsibilities, do not account appropriately for consultative, nighttime, and weekend work, and/or lack transparency may contribute to medical errors [13, 14], professional dissatisfaction [15], and neonatologist burnout [16, 17]. It is imperative to patient and workforce safety that NICUs staff appropriately. Although most trainees and non-physician members of the multidisciplinary NICU team have standard and/or maximum work hours defined by professional organizations, attending neonatologists do not currently enjoy such protection. Recently published staffing guidelines for neonatologists delineate consensus recommendations to ensure patient safety and workforce sustainability beyond annual hourly limits, including limitations on shift length, limitations on the amount of night work, and basic protections for periods when the available workforce exceeds clinical needs [9]. These guidelines are critically important, but do not provide guidance to clinical and/or administrative leaders on *how* to implement workforce limitations across the variety of roles and care models existing in modern neonatology. A transparent scheduling model that accounts for all clinical and non-clinical work is an important tool, as well as a guardrail to protect patients and physicians.

A transparent method to allot time is critical to meeting all institutional missions. Without appropriately dedicated resources, no mission, whether clinical, scholarly, or administrative, can be adequately accomplished. Staffing models that assume a clinical schedule equivalent to a 40–50 h work week *in addition* to unit management, scholarly activities, clinical teaching and mentorship, research, and administrative responsibilities are unsustainable, lead to poor quality of care and medical errors, physician exhaustion, and burnout that are not considered acceptable in other professions [13, 16, 17]. An explicit, transparent scheduling model allows for recognition of all types of professional responsibility and provides clarity for both attending neonatologists and upper-level administration regarding reasonable expectations of time for clinical and non-clinical work. This is imperative for 24-7 specialties such as neonatology, where the typical human resources model of weekday-only time credit is inappropriate and where many nighttime and weekend activities go unrecognized. Finally, a scheduling model that allows for the great diversity of clinical activities in neonatology allows for equity in scheduling, which is fundamental for the success of any group practice.

Other scheduling models used in neonatology and similar critical care settings have also been proposed. Traditional 12-h shift models commonly used in adult critical care and emergency medicine may be less well-suited for high-acuity NICUs due to the longitudinal nature of patient care over weeks to months in many cases. Schedules based on “weeks of service” often do not account for nighttime or weekend work, leading to physician burnout and safety concerns. Schedules based on point systems [18] are often popular with physicians but difficult for administrators to understand.

In our model, a 1.0 FTE academic neonatologist provides a maximum of 0.8 cFTE to allow the opportunity to pursue scholarly work fundamental to the academic mission. Those with additional responsibilities, such as a medical director, division director, or fellowship program director role, are allotted an additional 0.1–0.6 protected FTE depending on the scope of the role and regulatory requirements [19]. Although American College of Graduate Medical Education (ACGME) standards are available for fellowship program director positions, there is marked variation in the allotted time credit for division directors and medical directors, with little to no consideration of their scope of work. Additional protected time can be assigned at the discretion of division leadership to ensure that all institutional missions – research, education, clinical quality, etc. – are realistically achievable. In this model, less desirable roles or shifts can be valued more highly than weekday daytime hours to reflect the excess mental and physical burden and to incentivize physician willingness to serve; this assignment of hourly value allows for the desirable aspects of a point-based schedule while still presenting a time-based schedule familiar to the administrative team.

## Conclusion

Although other solutions exist (see Supplementary Tables 2 and 3 for examples), we share our solution to the challenges of complex neonatology staffing with neonatology leaders wishing to implement an equitable, transparent hours-based scheduling model.

## Supplementary information

Supplementary material

Supplementary Table 2

Supplementary Table 3
