# Supplementary material for: Hours based scheduling in neonatology: a practical approach
Source: J Perinatol. 2025 Jun 19;46(2):315–20. doi: 10.1038/s41372-025-02332-y (PMC12909100; doi:10.1038/s41372-025-02332-y)
Supplement: Supplementary file 1 — Supplementary material [file 41372_2025_2332_MOESM1_ESM.docx]

Supplemental Table 1. Clinical roles commonly covered by neonatologists

| **Role** | **Setting** |
| --- | --- |
| ICU coverage (level II, III, IV) | Inpatient and/or remote supervision, 24 hr/day |
| Delivery room coverage | Inpatient and/or remote supervision, 24 hr/day |
| Newborn nursery coverage (level I) | Inpatient and/or remote supervision, 24 hr/day |
| Neonatal transport (ground, air) | Inpatient and/or remote supervision, 24 hr/day |
| Prenatal consultation | Inpatient and/or ambulatory, and/or telehealth, 24 hr/day |
| Ambulatory follow-up programs | Ambulatory, weekday |
| Specialty services: |  |
| ECMO, CRRT | Inpatient and/or remote supervision, 24 hr/day |
| POCUS, Hemodynamics | Inpatient and/or remote supervision, hours vary by site |
| Neuro, cardiac critical care teams | Inpatient and/or remote supervision, 24 hr/day |
| Consultation (in-hospital; prior to transport) | Inpatient, telehealth, and/or remote supervision, 24 hr/day |

Supplemental Table 2. A second example of a CARTS-based neonatology schedule.^20^

In this schedule, 0.8 cFTE equates to 1650 hours. The method of allocation of clinical services is commonly referred to as the CARTS model or “one-minus” methodology, where 1.0 full-time equivalent (FTE) is defined, and all other non-clinical activities and responsibilities are subtracted to derive the clinical cFTE. For example, a clinician who has no other academic responsibilities and whose cFTE is 0.8 will be expected to complete 1650 hours.

Specific administrative portfolios have effort subtracted from this 1.0 cFTE baseline, e.g. 0.2 cFTE for division director, 0.1 cFTE for medical director. All portfolios that have administrative requirements (Neurocritical care director, quality lead, congenital diaphragmatic hernia lead, fellowship director, high-risk follow up director), received non-clinical FTE regardless of whether departmental/hospital funding is provided at the discretion of the division director. Non-clinical FTE credit is provided for funded research time. Once the various credits are removed the remaining hours are the expected clinical time. This is allocated as follows: rounding service and hemodynamics consultation service count for 70 hours / week. Other consultation work (e.g. BPD, ECMO, neurocritical care) is allocated as a projected average based on previous work. Night call is considered 1:1.

One of the exceptions to this rule is that junior physician scientists should complete a minimum of 8 weeks service to ensure they develop the required skills to become a neonatal intensivist. In addition, there are some faculty whose cFTE is zero (or close to it) who choose to continue clinical work to maintain their skills.

Supplemental Table 3. A third example of a CARTS-based neonatology schedule.^20^

In this model, a “year” is defined as 2,000 total hours as the basis of a shift-based model. The model uses a modified cFTE valuation system and the calculations that support the cFTE assignment. The institution’s neonatologists worked together to define the hours equivalency. A faculty-led scheduling committee reviews all FTE valuations annually to determine if updates are needed and to introduce recommendations for changes based upon 1) patient-centeredness, 2) transparency, and 3) equity. For example, the committee recently uncoupled weekends from weeks of service assignments to alleviate inequities for those with additional protected time compared those who are 90% clinical FTE. Faculty can select the services through which they achieve their assigned cFTE – and can trade calls/weekends to personalize their own schedule.
