# Supplementary material for: Hours based scheduling in neonatology: a practical approach
Source: J Perinatol. 2025 Jun 19;46(2):315–20. doi: 10.1038/s41372-025-02332-y (PMC12909100; doi:10.1038/s41372-025-02332-y)
Supplement: Supplementary file 2 — Supplementary Table 2 [file 41372_2025_2332_MOESM2_ESM.docx]

| **Faculty** | **Department cFTE** | **Divisional modifications cFTE** | **Divisional Roles** | **cFTE (Hrs)** | **Other Clinical Resp. (Hrs)** | **Adjusted Clinical Resp (hrs)** | **cFTE less Other Clinical Resp** | **Rounding Teams weeks (10 hrs/d)** | **Assigned we** | **Weeks diff** | **NCCU 1 mini** | **NCCU 2mini** | **Education Te** | **NNP Team** | **L6** |
| --- | --- | --- | --- | --- | --- | --- | --- | --- | --- | --- | --- | --- | --- | --- | --- |
|  | 0.8 | 0.7 | 0.1 (QI) | 1444 | 433 | 1011 | 0.49 | 14.4 | 14 | -0.4 | 4 | 2 | 4 | 2 | 2 |
|  |  | 0.8 |  | 1650 | 848 | 802 | 0.39 | 11.45 | 12 | 0.55 | 4 | 2 | 2 | 2 | 2 |
|  | 0.4 | 0.2 | 0.25 VC faculty devlel | 413 | 0 | 413 | 0.20 | 5.89 | 5 | -0.9 |  |  |  |  | 5 |
|  | 0.14 | 0.14 |  | 289 | 195 | 94 | 0.05 | 1.33 | 6 | 4.67 |  | 4 |  | 2 |  |
|  | 0.2 | 0.2 |  | 413 | 261 | 152 | 0.07 | 2.16 | 8 | 5.84 | 4 | 2 |  | 2 |  |
|  |  | 0.8 |  | 1650 | 835 | 815 | 0.40 | 11.64 | 12 | 0.36 | 4 | 2 | 2 | 2 | 2 |
|  | 0.55 | 0.55 |  | 1134 | 223 | 911 | 0.44 | 13.01 | 12 | -1 | 2 | 2 |  | 4 | 4 |
|  | 0.45 | 0.45 | 0.11 Med director | 928 | 80 | 848 | 0.41 | 12.1 | 12 | -0.1 |  |  | 6 |  | 6 |
|  | 0.68 | 0.66 | 0.02 (f/u director) | 1361 | 526 | 835 | 0.40 | 11.9 | 12 | -0.1 |  | 2 | 2 | 4 | 4 |
|  | 0.7 | 0.68 | 0.12 (APD, wellness, s | 1403 | 386 | 1017 | 0.49 | 14.52 | 14 | -0.5 | 4 | 2 | 4 | 2 | 2 |
|  | 0.74 | 0.71 | 0.03 (ECMO lead) | 1506 | 768 | 738 | 0.36 | 10.5 | 10 | -0.5 | 4 | 2 | 2 | 2 |  |
|  |  | 0.8 |  | 1650 | 706 | 944 | 0.46 | 13.4 | 14 | 0.6 | 4 | 2 | 4 | 2 | 2 |
|  | 0.6 | 0.6 | 0.2 ACMIO/div inform | 1238 | 315 | 923 | 0.45 | 13.1 | 12 | -1.1 | 2 | 4 | 2 | 2 | 2 |
|  | 0.78 | 0.75 | 0.05 (NCC lead) | 1547 | 821 | 726 | 0.35 | 10.36 | 10 | -0.36 | 2 | 4 | 2 |  | 2 |
|  | 0.57 | 0.47 | 0.1 (QI) | 969 | 63 | 906 | 0.44 | 12.94 | 12 | -0.94 |  |  | 4 | 4 | 4 |
|  | 0.01 | 0.01 | 0.45 div dir, VC crit ca | 21 | 272 | -251 | -0.12 | -3.6 | 4 | 7.6 | 2 |  |  | 2 |  |
|  | 0.38 | 0.38 | 0.53 res APD, fellow P | 784 | 256 | 528 | 0.26 | 7.5 | 8 | 0.5 |  | 4 | 2 |  | 2 |
|  | 0.6 | 0.6 |  | 1238 | 485 | 753 | 0.36 | 10.75 | 10 | -0.75 | 3 | 2 | 4 | 1 |  |
|  | 0.6 | 0.5 | 0.1 (HD lead) | 1031 | 377 | 654 | 0.32 | 9.3 | 8 | -1.3 |  | 4 | 2 | 2 |  |
|  | 0.65 | 0.65 | 0.1 med student rese | 1341 | 157 | 1184 | 0.57 | 16.9 | 12 | -4.9 |  |  |  | 6 | 6 |
|  |  | 0.2 |  | 413 | 286 | 127 | 0.06 | 1.8 | 8 | 7.2 | 4 | 2 |  | 2 |  |
|  | 0.8 | 0.75 | 0.05 (HD outpt) | 1547 | 845 | 702 | 0.34 | 10.02 | 10 | -0.02 | 3 | 2 | 4 |  | 1 |
|  | 0.76 | 0.6 | 0.2 (assoc med director/lung team  lead) | 1238 | 420 | 818 | 0.40 | 11.6 | 11 | -0.6 | 2 | 4 |  | 3 | 2 |
|  | 0.62 | 0.57 | 0.05 circs 0.19 (dir ad | 1176 | 304 | 872 | 0.42 | 12.45 | 12 | -0.45 |  | 2 | 4 | 4 | 2 |
|  | 0.8 | 0.77 | 0.03 CDH lead | 1588 | 711 | 877 | 0.43 | 12.53 | 12 | -0.53 | 4 | 2 | 2 | 2 | 2 |
|  |  |  |  |  |  |  |  |  | 260 |  | 52 | 52 | 52 | 52 | 52 |

| 14 |
| --- |
| 12 |
| 5 |
| 6 |
| 8 |
| 12 |
| 12 |
| 12 |
| 12 |
| 14 |
| 10 |
| 14 |
| 12 |
| 10 |
| 12 |
| 4 |
| 8 |
| 10 |
| 8 |
| 12 |
| 8 |
| 10 |
| 11 |
| 12 |
| 12 |

5x52=260 weeks
