# Supplementary material for: Hours based scheduling in neonatology: a practical approach
Source: J Perinatol. 2025 Jun 19;46(2):315–20. doi: 10.1038/s41372-025-02332-y (PMC12909100; doi:10.1038/s41372-025-02332-y)
Supplement: Supplementary file 3 — Supplementary Table 3 [file 41372_2025_2332_MOESM3_ESM.docx]

| **Neonatal Intensivist FTE Calculator 2000 hours/year (Shift-based)** | | | | | | |
| --- | --- | --- | --- | --- | --- | --- |
|  | | | | | | |
|  | **Protected FTE (pFTE)** | | |  | | |
|  |  | **Base pFTE (0.1)** | **0.10** |  | | |
|  |  | **Research pFTE** | **0.00** |  | | |
|  |  | **Administrative pFTE** | **0.00** |  | | |
|  |  | **Total pFTE** | **0.10** |  | | |
|  |  | | |  | | |
|  | **Clinical FTE (cFTE)** | | |  | | |
|  | **Shared cFTE (all 1.0 FTE have this base contribution)** | | |  | | |
|  | **Holiday Week Backup/Consults (Payback)** | **cFTE # Weeks (7d)**  **0.05 1**  **0.02 2** | **Total 0.05**  **0.04** | 12 hours/day x 4 days + 8 hours/day x 3 days = 72 hours / 2000 hours = 0.04 + 0.01 bonus credit for holiday) = 0.05  10 hours/day x 7 days = 70 hours / 2000 hours = 0.04 (x 0.5 adjustment factor) = 0.02 | | |
|  | **Weekends (NICU Level 4) Weekends (Level 2)**  **Weeknight Call Weekend/Holiday Call** | **# Weekends (12 total)**  **0.01 6**  **0.01 6**  **# Calls**  **0.01 8**  **0.02 4** | **0.06**  **0.06**  **0.08**  **0.08** | 8 hours/day x 2 days = 16 hours / 2000 hours = 0.01  24 hours/day x 2 days = 48 hours / 2000 hours = 0.02 (x 0.5 Adjustment factor for volume/acuity) = 0.01  16 hours/call / 2000 hours = 0.008 (x 1.5 Adjustment factor for volume/acuity/night) = 0.01  20 hours/call / 2000 hours = 0.01 (x 2.0 Adjustment factor for volume/acuity/weekend/holiday shift) = 0.02 | | |
|  |  | **Total Shared cFTE** | **0.37** |  | | |
|  | **Individual cFTE (faculty preference)** | | |  | | |
|  | **NICU Level 4** | **cFTE # Weeks (5d)**  **0.03 0** | **Total**  **0.00** |  | | |
|  |  |  |  |  | 12 hours/day x 5 days = 60 hours / 2000 hours = 0.03 |  |
|  | **NICU Level 2 (24 h)**  **ECMO (24 h)** | **0.03 0**  **# Weeks (7d)**  **0.02 0** | **0.00**  **0.00** |  | 24 hours/day x 5 days = 120 hours / 2000 hours = 0.06 (x 0.5 Adjustment factor for volume/acuity) = 0.03 |  |
|  |  |  |  | 24 hours/day x 7 days = 168 hours / 2000 hours = 0.08 (x 0.5 Adjustment factor) = 0.04 | | |
|  | **Clinics** | **cFTE # Weeks (5d)**  **0.02 0** | **Total 0.00** | 8 hours/session x 5 days = 40 hours / 2000 hours = 0.02 | | |
|  |  | **Total Individual cFTE**  **Total cFTE** | **0.00**  **0.37** |  | | |
|  | | | | | | |
|  |  | **Total FTE (1.0)** | **0.47** |  |  |  |
